# Phytochemical Profile and Antioxidant Capacity of Coffee Plant Organs Compared to Green and Roasted Coffee Beans

**DOI:** 10.3390/antiox9020093

**Published:** 2020-01-22

**Authors:** Robert Acidri, Yumiko Sawai, Yuko Sugimoto, Takuo Handa, Daisuke Sasagawa, Tsugiyaki Masunaga, Sadahiro Yamamoto, Eiji Nishihara

**Affiliations:** 1 The United Graduate School of Agricultural Sciences, Tottori University, 4–01 Koyama-cho Minami, Tottori 680-8553, Japan; acidrirobert24@gmail.com (R.A.); actias.luna0530@gmail.com (T.H.); ssgw.2653.02.06@gmail.com (D.S.); 2 Sawai Coffee Limited, 278–6, Takenouchi danchi, Sakaiminato City, Tottori 648–0046, Japan; yumiko@sawaicoffee.co.jp; 3 Tottori Institute of Industrial Technology, 2032–3, Nakano-cho, Sakaiminato-shi, Tottori 684–0041, Japan; sugimoto-yu@pref.tottori.lg.jp; 4 Faculty of Soil Eco-Engineering and Plant Nutrition, Shimane University, 1060, Nishikawatsucho, Matsue 690-8504, Japan; masunaga@life.shimane-u.ac.jp; 5 Faculty of Agriculture, Tottori University, 4–101 Koyama-cho Minami, Tottori 680-8553, Japan; yamasada@tottori-u.ac.jp

**Keywords:** alkaloids, antioxidant capacity, carotenoids, chlorophylls, *Coffea arabica* L., phenolic compounds, sucrose

## Abstract

The current study investigates the phytochemical composition of coffee plant organs and their corresponding antioxidant capacities compared to green and roasted coffee beans. HPLC analysis indicated that the investigated compounds were present in all organs except mangiferin, which was absent in roots, stems and seeds, and caffeine, which was absent in stems and roots. Total phytochemicals were highest in the green beans (GB) at 9.70 mg g^−1^ dry weight (DW), while roasting caused a 66% decline in the roasted beans (RB). This decline resulted more from 5–CQA and sucrose decomposition by 68% and 97%, respectively, while caffeine and trigonelline were not significantly thermally affected. Roasting increased the total phenolic content (TPC) by 20.8% which was associated with an increase of 68.8%, 47.5% and 13.4% in the antioxidant capacity (TEAC) determined by 2,2–diphenyl–1–picryl hydrazyl radical (DPPH), 2,2–azino bis (3–ethyl benzothiazoline–6–sulphonic acid) radical (ABTS) and Ferric ion reducing antioxidant power (FRAP) assays, respectively. Amongst the leaves, the youngest (L1) contained the highest content at 8.23 mg g^−1^ DW, which gradually reduced with leaf age to 5.57 mg g^−1^ DW in the oldest (L6). Leaves also contained the highest TPC (over 60 mg g^−1^ GAE) and exhibited high TEAC, the latter being highest in L1 at 328.0, 345.7 and 1097.4, and least in L6 at 304.6, 294.5 and 755.1 µmol Trolox g^−1^ sample for the respective assays. Phytochemical accumulation, TPC and TEAC were least in woody stem (WS) at 1.42 mg g^−1^ DW; 8.7 mg g^−1^ GAE; 21.9, 24.9 and 110.0 µmol Trolox g^−1^ sample; while herbaceous stem (HS) contained up to 4.37 mg g^−1^ DW; 27.8 mg g^−1^ GAE; 110.9, 124.8 and 469.7 µmol Trolox g^−1^ sample, respectively. Roots contained up to 1.85 mg g^−1^ DW, 15.8 mg^−1^ GAE and TEAC of 36.8, 41.5 and 156.7 µmol Trolox g^−1^ sample. Amongst the organs, therefore, coffee leaves possessed higher values than roasted beans on the basis of phytochemicals, TPC and TEAC. Leaves also contain carotenoids and chlorophylls pigments with potent health benefits. With appropriate processing methods, a beverage prepared from leaves (coffee leaf tea) could be a rich source of phytochemicals and antioxidants with therapeutic and pharmacological values for human health.

## 1. Introduction

Coffee remains the most valuable primary product globally, employing over 26 million people along the chain from cultivation mainly in developing countries to consumption in developed countries [1]. By 2017, 160 million bags of coffee were exported, of which arabica (*Coffea arabica* L.) accounted for 63.3% of the total and the rest being mainly robusta (*C. canephora* Pierre ex A. Froehner). It has been estimated that the coffee industry value is over 100 billion USD worldwide with up to 20 billion USD involved in export alone. Coffee is mainly traded as raw or processed beans. Therefore, it is not surprising to give considerable attention to the fluctuations in the phytochemical profile that occur during fruit growth and maturation [2,3,4,5,6], as well as changes induced during processing such as fermentation and roasting of the coffee beans after harvest [7].

In mature coffee beans, accumulation of sucrose, caffeine, chlorogenic acids and trigonelline has been well investigated due to the involvement of these compounds in flavour formation and the characteristic bitter taste of the coffee beverage [7,8]. Sucrose and its derivatives fructose and glucose provide the carbonyl group that combines with the hydrazyl group from amino acids to form a number of odorants during caramelisation in Strecker and Maillard reactions [8]. The thermal degradation of trigonelline into pyrroles and pyridine derivatives has also been reported to contribute to coffee aroma [9,10]. On the other hand, products of chlorogenic acids thermal degradation as well as caffeine, which is barely affected by high temperatures during roasting, are responsible for the characteristic bitter taste of the beverage [11]. Moreover, the ratio of chlorogenic acids to caffeine has also been described as an important parameter that might have an effect on taste, antioxidant potential and preference of a given type of beverage [12].

On the other hand, previous reports have suggested that coffee leaves may contain all the major metabolites present in the seeds [6]. In addition, they contain mangiferin, a xanthonoid with great antioxidant potential as well as therapeutic and pharmacological properties, and although present in the fleshy fruit parts, this compound is absent in the endosperm [13,14,15]. As a result, the use of coffee leaves as a dietary source of antioxidants in form of “coffee leaf tea” might become more common. Moreover, several processing methods like those used in tea processing have since been suggested [16]. Coffee leaves also contain carotenoids and chlorophyll pigments whose antioxidant value for dietary intake can be harnessed by using appropriate processing methods such as in green tea processing [16,17,18,19]. It has been demonstrated that food processing generally affects the content of the phytochemicals and the corresponding antioxidant capacities in foods [20]. Until today, coffee beans and recently leaves are the main sources of antioxidants from coffee plants. These undergo either roasting or some form of drying before utilisation. The two processes may result in significant changes in the biochemical composition and/or antioxidant capacities of the resultant beverage [7,16]. For example, Strecker and Maillard reaction may increase oxidant scavenging abilities due to the formation of new compounds [21].

Although it is likely that other organs of the coffee plant such as roots or stems may accumulate similar phytochemicals, their utilisation and/or investigations have not received similar attention. In fact, only a few reports have elucidated accumulation of some alkaloids and phenolic compounds in other organs such roots of seedlings or juvenile coffee plants [22,23]. Moreover, reports on the accumulation of the major phytochemicals in all coffee plant organs in comparison with the content in green and roasted coffee beans are still lacking. In addition, studies comparing the relationship between this phytochemical composition and the antioxidant capacities in the organs to the typical dietary antioxidant sources (beans and leaves) remain uninvestigated whatsoever. Similarly, phytochemical changes with corresponding effects on the antioxidants capacities, resulting from bean or leaf processing, still remain unclear.

This study therefore aims at assessing the phytochemical composition and antioxidant capacities of coffee plant organs such as coffee seeds, leaves, stems and roots. In addition, the changes induced by roasting and leaf senescence on the phytochemical composition and antioxidant capacities of beans and leaves, respectively, are investigated. Coffee is mainly consumed as a beverage processed from coffee beans; therefore, we also investigated whether other organs, especially leaves, might have high antioxidant activities and/or phytochemical content in relation to roasted coffee beans. Finally, relationships amongst plant organs in terms of phytochemical composition and antioxidant capacities are evaluated. 

## 2. Materials and Methods 

### 2.1. Seeds

Seeds of *C. arabica* were imported as dried depulped cherries (green beans) from Indonesia for commercial roasting by Sawai Coffee Limited, Tottori, Japan. From these, raw and roasted bean samples were obtained and used in the current study. Some of the raw seeds were germinated and raised into plants for further investigations. 

### 2.2. Plant Material and Growing Conditions 

The seeds were soaked in running water for 3 days and pre-germinated on moist paper towels in an incubator at 30 °C in the dark [24]. After sprouting, each seedling was transplanted into a 3-L pot containing a mixture of peat moss, perlite and humus at a ratio of 5:3:2, respectively, and grown in a naturally lit vinyl–house at temperatures above 20 °C. The seedlings were irrigated, first with half-strength nutrient solution for the first 6 months and then with the full-strength solution prepared according to Hoagland and Arnon [25], with a few modifications. The nutrient solution composition included in mmol/L, 5.7 N–NO_3_^−^, 1 N-NH_4_^+^, 0.1 P–H_2_PO_4_^−^, 2.4 K^+^, 1.2 Ca^2+^, 0.6 Mg^2+^, 0.7 S–SO_4_^2−^, and in µmol/L 35 Fe III EDTA, 0.8 Cu, 1.5 Zn, 5 Mn, 17 B, and 0.1 Mo. Coffee plants were grown for one and a half years from May 2017 until they attained 6 pairs of fully expanded leaves on the main stem.

### 2.3. Sample Preparation 

All the samples were thoroughly washed, rinsed with distilled water and immediately flash-frozen in liquid nitrogen before storing under −80 °C in a freezer awaiting further processing.

#### 2.3.1. Coffee Seeds

Both green seeds (GB) and roasted seeds (RB), characteristically known as green and roasted coffee beans, respectively, were obtained in triplicates and treated as independent samples. 

#### 2.3.2. Leaves

Each leaf pair was sampled independently and labelled from L1 (youngest) to L6 (oldest). In addition, to determine the phytochemical changes that occur after sun drying, naturally dried leaves corresponding to the first leaf pair were sampled from the dried plants and labelled as brown leaves (BL).

#### 2.3.3. Stems

The stem was divided in to two parts:

(1) The herbaceous stem (HS), consisting of the green parts of the main stem and the green branches.
(2) The woody stem (WS), consisting of the ligneous brown part of the stem above the root collar.

#### 2.3.4. Roots

Roots were thoroughly cleaned off of all the soils before further processing.

### 2.4. Extraction and Analysis 

#### 2.4.1. Chlorophylls and Carotenoids Determination

Leaf pigments were extracted with 2.5 mM sodium phosphate 80% buffered acetone (pH 7.8) and quantified spectrophotometrically (Hitachi ratio beam spectrophotometer U–500, Tokyo, Japan). Both pigments were extracted from about 0.25 g fresh leaf samples with liquid nitrogen and centrifuged at 29,300× *g*. Both chlorophylls and carotenoids were assayed according to Porra et al. [26] and determined using equations developed by Lichtenthaler and Buschmann [27]. 

#### 2.4.2. Sample Processing for Phytochemical and Antioxidant Capacity Determination

All the samples were freeze dried at −12 °C (Eyela DRC 1000–FDU 1110, Tokyo, Japan) to a constant moisture content for 72 h. The dried samples were milled into a fine powder using a wonder blender (Osaka chemical Co., Ltd., Osaka, Japan). All the processed samples were kept in airtight self-seal poly bags with silica gel awaiting extraction and analysis. 

#### 2.4.3. Sample Extraction

Extraction proceeded as described by Chen [16], with a few modifications. The sample powders were infused in 25 mL boiling water in 50 mL Falcon conical centrifuge tubes (Thermo Fisher Scientific, MA, USA) for 8 min. The samples were thereafter cooled under room temperature then centrifuged at 29,300× *g* at 25 °C for 15 min. The supernatants were collected in 50 mL volumetric flasks and to the residue was added 10 mL cold water for re-extraction. For each sample, all the supernatants were pooled together, volumetric flasks filled to the mark and the extracted samples then filtered using 0.45 µm Millipore (Sigma and Aldrich, Tokyo, Japan) into 50 mL glass vials. From each sample, an aliquot of about 2 mL was obtained for HPLC phytochemical analysis. The extracted samples in the glass vials were freeze dried at −20 °C (Eyela DRC 1000–FDU 1110, Tokyo, Japan) for 7 days. Extraction yield was calculated as the difference in the weights between the sample powder and the extracted freeze-dried powder, multiplied by 100.

#### 2.4.4. Alkaloids and Phenolic Compounds Determination

Alkaloids and phenolic compounds were determined simultaneously using a high–performance liquid chromatography (HPLC) system equipped with a UV detector at a wavelength of 270 nm (Hitachi L–2490, Hitachi, Tokyo, Japan) from a 10 µL sample extract. Separation of the analytes was performed on a TSKgel ODS–100 C18 column (5 µm particles size, 4.6 × 150 mm) in a thermostatic oven at a temperature of 40 °C (Sigma–Aldrich, Tokyo, Japan) with a binary phase mobile gradient at a total flow rate of 0.4 mL min^−1^. The mobile phase consisted of two filtered (0.22 µm Millipore), sonicated and degassed solvents A (methanol, 100%) and B (acetic acid: H_2_O, 98:2, *v/v*) with linear evolution of the gradient profile according to a set elution program (Table 1). Calibration curves were obtained from three replicate points for the standard compounds (trigonelline, 5–caffeoylquinic acid, caffeine and mangiferin). Each analyte from the samples was then identified by peak position and thereafter quantified by peak area measurement using regression equations developed from calibration curves for the standard compounds. All the analytical standards and the organic solvents used were of HPLC grade (Sigma–Aldrich, Tokyo, Japan).

#### 2.4.5. Sucrose Extraction and Determination

Extraction was done from 0.5 g freeze-dried powder in Falcon tubes using 30 mL of ultrapure water. The contents were sonicated for 30 min, centrifuged at 29,300× *g*. Extraction was repeated 3 more times and all the supernatants pooled together. The final extract was filtered (0.22 µm) and thereafter, all the solvent evaporated off using a rotary evaporator (Rotavapor R–300 Buchi, Flawil, Switzerland). The residue was redissolved, first in 3 mL ultrapure water and then to this solution was added 3 mL acetonitrile solvent. The new solution was re-filtered (0.22 µm) and analysed using HPLC. Sucrose content was determined using the same HPLC system described above but with an RI detector at a temperature of 35 °C and a shodex (R spak DC–613) column (5 µm, 150 mm × 4.6 µm made by Sigma–Aldrich, Tokyo, Japan) at a temperature of 55 °C. The flow liquid consisted of acetonitrile and water at 75% and 25% (*v/v*), respectively. Elution was done at a flow rate of 1.0 mL min^−1^, resulting into a retention time of 9.30 min for sucrose standards and the samples. Other sugars such as glucose and fructose were undetectable due to their low concentration in the samples. 

#### 2.4.6. Total Phenolic Content (TPC) Determination 

Total phenolic content was determined from 0.5 mL of the collected aliquots described in Section 2.4.3 according to Chen [28], with slight modifications. Each sample was diluted 10–fold from which 1 mL was transferred to a new test tube. The samples and/or gallic acid standards were then incubated with 5 mL of 10% Follin–Ciocalteu’s reagent for one minute and then reacted with 4 mL of 20% (*w/v*) sodium carbonate solution for 30 min at room temperature. Absorbance was then read at 765 nm using the Hitachi ratio beam spectrophotometer.

#### 2.4.7. Antioxidant Capacity Determination

The ability of coffee sample extracts to scavenge reactive species was determined by dissolving the freeze-dried powder in ultrapure water followed by dilution with methanol, as described by Chen [16]. Each sample was divided into 4 subsets of serial dilutions and stored under −80 °C until further analysis. 

##### 2,2–Diphenyl–1–picryl hydrazyl (DPPH) Radical Assay

The DPPH radical was used to determine the free radical scavenging capacity of the coffee sample extracts following procedures described by Chen [16]. For analysis, the frozen samples were adjusted to room temperature, from which 1 mL was transferred to a new test tube, diluted 10–fold with methanol and then incubated with freshly prepared 0.1 mmol/L DPPH solution for 10 min in the dark in a total volume of 10 mL. In addition, freshly prepared Trolox (6–hydroxy–2,5,7,8–tetramethyl–chroman–2–carboxylic acid) standards of serial concentrations were treated in the same way as the samples. Absorbances of both the standard and samples were determined at 519 nm using the Hitachi ratio beam spectrophotometer. The concentration that caused a 50% decrease in the initial concentration of the DPPH radical defined as IC_50_ was determined for both the standard and the samples from the percentage inhibition of the DPPH radical which was calculated as % inhibition = (Abs_control_ − Abs_sample_)/(Abs_control_ − Abs_blank_) × 100 where Abs_control_ = absorbance of the 0.1 mmol/L DPPH methanol solution, Abs_sample_ = absorbance of the 0.1 mmol/L methanol solution after fading induced by addition of sample or Trolox, and Abs_blank_ = absorbance of the methanol solvent only. Antioxidant capacity of the samples, as determined using the DPPH scavenging radical, was calculated as (IC_50 (Trolox)_/IC_50 (sample)_) × 10^5^ and expressed as µmol Troloxg^−1^ sample of Trolox equivalent antioxidant capacity (TEAC). 

##### 2,2–Azino bis (3–ethyl benzothiazoline–6–sulphonic acid) Radical (ABTS) Assay

The ABTS radical scavenging capacity was determined, as modified by Chen [16] with a few changes. ABTS solution of 7 mmol/L was prepared and mixed with K_2_S_2_O_8_ solution of 140 mmol/L at a ratio of 5 mL:88 µL, respectively. The solution was then incubated at room temperature in the dark overnight. On the ABTS measurement day, the stock solution was diluted with ultra-pure water at a ratio of 0.6:40 mL, respectively, and the absorbance adjusted to 0.7 ± 0.02 at 734 nm by spectrophotometry. Serial dilutions of freshly prepared Trolox standard and coffee sample extracts of 0.5 mL were then incubated with 9.5 mL of the new solution for 10 min at room temperature in the dark after which absorbance was read at the same wavelength. ABTS antioxidant capacity was then calculated as gradient of Trolox standard/ gradient of sample and expressed as µmol Troloxg^−1^ sample of TEAC.

##### Ferric Ion Reducing Antioxidant Power (FRAP) Assay 

The FRAP assay was carried out with the following procedures modified by Alvarez-Jubete [20]. The FRAP oxidant solution consisted of 0.2 M sodium acetate buffer (pH 3.6), 20 mM ferric chloride solution and 10 mM TPTZ (2,4,6 tris (2–pyridyl)–s–triazine) solution in 40 mM HCl mixed at a ratio of 10:1:1, respectively. The serially diluted samples and Trolox standard of 0.5 mL were incubated with 9.5 mL of freshly prepared FRAP oxidant solution in a water bath at 37 °C for 40 min after which absorbance was read at 593 nm. The FRAP antioxidant capacity was then calculated as the gradient of Trolox standard/gradient of sample and expressed as µmol Troloxg^−1^ sample of TEAC.

### 2.5. Statistical Analysis 

Experimental data were analysed using Stata 12.0 statistics and data analysis program (Stata Corp, College Station, TX, USA). Group means of the parameters were analysed using one-way analysis of variance (ANOVA) and compared for statistical significance using Tukey’s test at *p* ≤ 0.05. Correlations and relationships amongst the parameters and amongst the plant organs were evaluated using Pearson’s correlation coefficient and principal component analysis (PCA) in Minitab v14, Minitab Inc. (State College, PA, USA). All analyses were performed in triplicates and data are expressed as mean ± S.D, *n* = 3.

## 3. Results

### 3.1. HPLC Phytochemical Composition 

**Figure 1:**
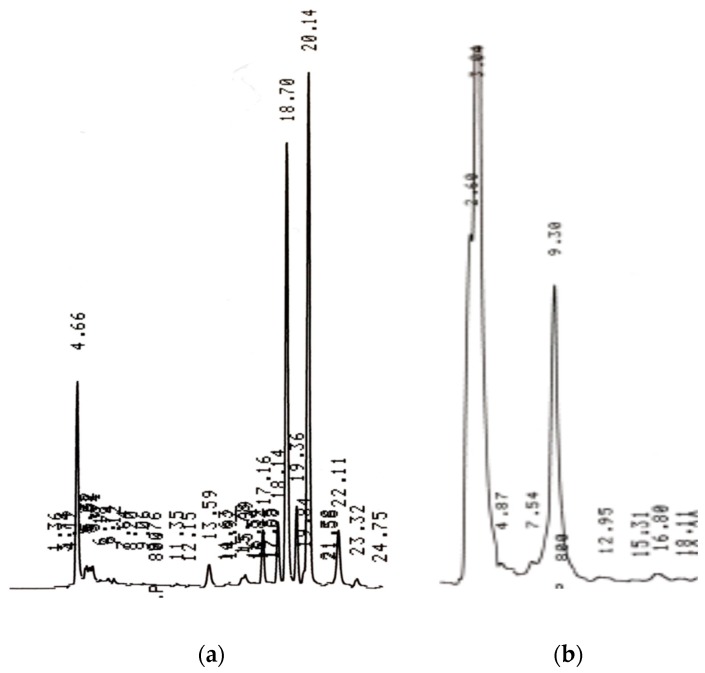
High–performance liquid chromatography (HPLC) chromatograms of the extracted coffee sample. (**a**) Displaying peaks for trigonelline, 5–CQA, caffeine and mangiferin at 4.66, 18.70, 20.14 and 22.11 min, respectively, obtained using an ultra–violet ( UV) detector at 270 nm; (**b**) displaying sucrose peak at 9.30 min obtained using a refractive index (RI) detector.

Typical HPLC chromatograms of the samples displaying the coeluted compounds (trigonelline, 5–CQA, caffeine and mangiferin) detected under UV and sucrose detected under RI are shown in Figure 1a,b, respectively. 

#### 3.1.1. Coffee Beans

The HPLC phytochemical profile of coffee beans was significantly different between GB and RB (Table 2). This pronounced difference was caused more by changes in sucrose and 5–CQA content, which were also the most abundant compounds in the raw beans. On the other hand, due to the degradation of sucrose and 5–CQA during roasting, the two alkaloids, caffeine and trigonelline, were the most abundant in RB. In fact, roasting reduced the total content of the phytochemicals by 66%. Moreover, the HPLC profile revealed that mangiferin was absent in the GB and definitely in RB.

#### 3.1.2. Leaves 

The HPLC phytochemical profile of the leaves indicated in Table 2 revealed that the most abundant isomer of chlorogenic acid (5–CQA) was the most abundant phytochemical determined, while mangiferin was the least contained in the coffee leaves. 5–CQA, sucrose, trigonelline and mangiferin were highest in the youngest leaves at 3.97, 2.63, 0.67, and 0.09 mg g^−1^ DW, respectively. These phytochemical compounds were accordingly least in the oldest leaves 2.48, 1.51, 0.47 and 0.06 mg g^−1^ DW, respectively. On the contrary, caffeine accumulated more with an increase in leaf age and therefore was highest in the oldest leaves compared to the youngest ones at 1.05 and 0.85 mg g^−1^ DW, respectively. Nevertheless, the youngest leaves contained the highest amount of the total phytochemicals analysed in the current study at 8.23 mg g^−1^ DW, while the oldest leaves contained the least at 5.57 mg g^−1^ DW. Moreover, the ratio of 5–CQA to caffeine also reduced gradually with increasing leaf age from 4.58 to 2.36 in the youngest and the oldest leaves, respectively. There was a decline in the content of the phytochemicals induced by leaf senescence, as observed in BL compared to L1. Trigonelline content was least affected by senescence despite reducing by around 50%. The content of caffeine, 5–CQA and sucrose reduced by over 60%, while mangiferin was completely degraded by senescence. On the other hand, there was an increase in the ratio of 5–CQA to caffeine due to a more pronounced decline in the content of caffeine than that of 5–CQA, 63% and 61%, respectively. 

The concentrations of chlorophylls and carotenoids in the leaves are indicated in Table 3. The concentration of chlorophyll *a*, total chlorophylls and total carotenoids showed no significant differences (*p* ≤ 0.05) with leaf age. On the other hand, that of chlorophyll *b* tended to increase as the leaves mature hence resulting in an increase in the chlorophyll *a*/*b* ratio in the older leaves. Contrastingly, although the ratio of total chlorophylls to total carotenoids, which is an indicator of the status of the photosynthetic apparatus, tends to reduce in the older leaves, it showed no significant difference. 

#### 3.1.3. Stems

There was a dramatic variation in the content of the phytochemicals within the stem (Table 2). The herbaceous stem (HS) contained more phytochemicals compared to the WS. In fact, the current study revealed that the content of the phytochemicals decreased by over 70% when the herbaceous stem tissues were lignified into woody tissues. In addition to the total decline of caffeine and mangiferin, there was a decrease of 54%, 55% and 75% in the contents of sucrose, trigonelline and 5–CQA, respectively. The relatively higher content of 5–CQA in both HS and WS coupled with a low content of caffeine resulted in an amazingly high 5–CQA/caffeine ratio, with the ratio being highest in WS. 

#### 3.1.4. Roots 

Coffee roots accumulated all the phytochemicals determined by the HPLC as in other organs, with the exception of caffeine and mangiferin (Table 2). Sucrose was the highest at 1.01%, followed by 5–CQA at 0.71%, whereas trigonelline was least at 0.14%. 

### 3.2. Total Phenolic Content and Antioxidant Capacities of the Coffee Plant Organs

The extract yield, total phenolic content and antioxidant capacities of the different coffee organs showed great variation amongst the samples (Table 4). The current study revealed that RB and the youngest leaves (L1) had the highest extraction yield at 36.4% and 26.6%, respectively. This was followed by GB at 25.8% and older leaves at 25.6%, accordingly. The yield was least for HS, roots and WS at 16.9%, 14.6% and 6.4% respectively. Similarly, TPC of the extracts was highest in the leaves and, more so, increasing with an increase in leaf age from 65.1 to 71.5 mg g^−1^ GAE. In addition, senescence did not significantly affect the content of the total phenols in the youngest leaves despite showing a decline to 60.4 mg g^−1^ GAE in BL. In beans however, roasting caused an increase in total phenolic content of 20.8% from 29.3 to 35.4 mg g^−1^ GAE. The total phenolic content of the stems was low at 27.8 and 8.7 mg g^−1^ GAE in HS and WS, respectively, while that of roots was 15.8 mg g^−1^ (Table 4).

Similar to the variation in TPC, both leaves and beans had consistently the highest TEAC values as determined by DPPH, ABTS and FRAP and lowest DPPH IC_50_ values (Table 4). This is an indicator of higher antioxidant capacity in these samples. Nevertheless, within beans, there was a consistent increase in the antioxidant capacity when green beans were roasted and hence resulting in an increment of 68.8%, 47.5% and 13.4% in DPPH, ABTS and FRAP values, respectively. This was accompanied by lower IC_50_ values in RB compared to GB at 87.0 and 146.8 µg sample/mL, respectively. Despite the variation being statistically non-significant within the leaves, there was a consistent decrease in the antioxidant capacities induced by leaf maturity. The TEAC of the youngest leaves (L1) was 328.0, 345.7 and 1097.4, as determined by DPPH, ABTS and FRAP assays, respectively, while the same assays showed lesser values in the oldest leaves (L6) at 223.4, 208.8 and 624.1. The younger leaves (L2 to L5) consistently showed intermediate values of antioxidant capacity. In addition, senescence somewhat caused a consistent decline in the antioxidant capacity of the youngest leaves which was more perceptible in the FRAP TEAC values (31.2% decline) compared to 7.13% and 14.8% for DPPH and ABTS, respectively. Antioxidant capacity was least in WS, showing a remarkable increase of 80.3%, 80.0% and 76.6% in DPPH, ABTS and FRAP TEAC values, respectively in HS. This was accompanied by lower IC_50_ values in HS compared to WS at 263.8 and 1403.1, respectively (Table 4). The antioxidant capacity of roots was intermediate between WS and HS with a IC_50_ value of 916.1, while DPPH, ABTS and FRAP values were 36.8, 41.5 and 156.7 µmol Trolox g^−1^ root sample, respectively.

### 3.3. Relationships amongst Phytochemicals and Plant Organs

**Figure 2:**
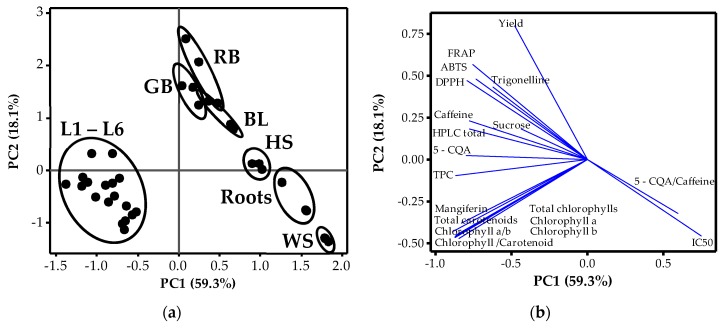
Principal component analysis summarizing the relationships among variables listed in Table 2, Table 3 and Table 4; (**a**) score plot of phytochemical contents according to plant organ; (**b**) loading plot of variables: sucrose, caffeine, trigonelline, 5–CQA, mangiferin, HPLC total, chlorophyll *a*, chlorophyll *b*, total chlorophyll, total carotenoids, chlorophyll *a*/*b*, chlorophyll/carotenoids and antioxidant capacity; DPPH, IC_50_, ABTS and FRAP. GB = green beans, RB = roasted beans, L1 to L6 = leaf pairs (1st–6th) from top to bottom of the coffee plant, BL = brown leaves of the first pair, HS = herbaceous stem, WS = woody stem.

Principal component analysis of the different coffee plant organs and 19 variables, including pigments, phytochemicals, TPC and antioxidant capacity, revealed that the two principal components explained 77.4% of the total variance in the data with a contribution of 59.3% and 18.1% for PC1 and PC2, respectively (Figure 2a,b). Principal Component 1 (PC1) clearly separated the samples into two groups based on the presence or absence of chlorophylls, carotenoids and mangiferin whereas PC2 separated the samples according to the content of the other phytochemicals, TPC and antioxidant capacities. The score plots for PC1 and PC2 revealed that RB, GB and BL contained high amounts of TPC and antioxidant capacities and hence loaded highest on PC2, followed by leaves (L1–L6), especially the youngest (L1) and then HS, respectively (Figure 2a). On the other hand, both roots and WS contained low amounts of caffeine and hence had a very high 5–CQA/caffeine ratio. Moreover, the accumulation of low phytochemicals and low TPC in these samples meant very low antioxidant capacities and very high IC_50_ values, hence positioning of these samples in the bottom right corner of the PCA chart. Variation amongst the leaves in terms of leaf pair positioning was less significant and therefore samples L1–L6 were clustered on the left-hand side of the PCA chart. This was due to the presence of chlorophylls, carotenoids and mangiferin, which were barely present in other samples. Similarly, these samples had relatively similar antioxidant capacities with corresponding DPPH IC_50_ values. Basing on PCA (Figure 2) and correlation coefficient results (Table S1), there was a significant positive correlation (*p* ≤ 0.01) amongst the different measures of the antioxidant capacity (DPPH, FRAP and ABTS). In addition, this antioxidant capacity correlated positively with TPC which also amongst the phytochemicals correlated more strongly with mangiferin and 5–CQA content. On the other hand, DPPH IC_50_ correlated negatively with both the antioxidant activities as wells as the phytochemical content in the plant organs. Amongst the phytochemicals, caffeine correlated more strongly with trigonelline whereas 5–CQA correlated more strongly with mangiferin (*p* ≤ 0.01). In addition, these phytochemicals correlated strongly with all the chlorophylls and carotenoids parameters. Nevertheless, the correlation between these pigments was most significant with mangiferin compared with the rest of the phytochemicals. 

## 4. Discussion

*Coffea* species contain several phytochemicals such as caffeine, trigonelline, chlorogenic acids, mangiferin, sucrose (Figure 1), which render their wide exploitation for pharmacological and health promoting benefits [6]. The utilization of coffee plants has mainly focused on a single organ, the coffee seeds, as the source of the phytochemicals with related health benefits [7]. However, utilization of other organs, including those evaluated in the current study, is also increasingly becoming common because of their possible therapeutic values [16]. Moreover, organs such as leaves have always been used traditionally in many coffee-growing regions for mitigation of a number of illnesses including cardiovascular, gastrointestinal, cancer, diabetes, dermatological and obesity amongst others [6]. In addition to containing all the known phytochemicals in the coffee seeds, the leaves contain exclusive compounds such as mangiferin (1,3,6,7-tetrahydroxyxanthone-C2-β-D-glucoside) and pigments like chlorophylls and carotenoids, whose antioxidant potency is also widely reported [13,14,15,16,17,18,19].

The current study revealed that raw beans contained the highest amounts of the phytochemicals, especially 5–CQA, sucrose, caffeine and trigonelline. Coffee seeds remain the most important organ in coffee trade because of their extensive use in the coffee beverage processing [7]. Phytochemicals in seeds accumulate as a result of metabolism within the fleshy parts of the fruits during maturation but also due to deposition, having been processed from leaves and young buds of the coffee plant [2,3,4,5]. Coffee seeds contain mainly carbohydrates; sucrose being the main constituent, whose role is to provide nourishment for the embryo in case the seeds germinate [29]. Also, similar to the findings in the current study (Table 2), high amounts of phenolic compounds have been reported in the coffee fruits and seeds. Chlorogenic acids are the main phenolic compounds that accumulate in the beans during the maturation of coffee fruits and seeds [2,5]. Of these, 5–CQA forms the main constituent of these hydroxycinnamic acid esters [14]. On the other hand, although mangiferin, another phenolic compound, was reported in the fruits, its accumulation in the seeds has been disputed [14], which explains its absence in all the beans in our study (Table 1). Phenolic compounds in the seeds and fleshy parts of the young coffee fruits are associated with their role in defence against oxidative stress and for future use in the synthesis of cell wall–bound phenolic polymers after seed germination and during seedling development [22]. However, as the fruits mature, a decline in the total chlorogenic acids content from between 5–7 mg g^−1^ DW by up to 7% or more occurs as a result of development of an elaborate enzymatic antioxidant system and reduced activities of polyphenol peroxidase and oxidase activities, hence warranting a lesser role of the phytochemicals in defence against reactive oxygen species [30,31]. In the current study, the content of 5–CQA in the mature GB was 3 mg g^−1^ DW (Table 2), which is consistent with the above findings. Before utilization as a beverage, coffee beans normally undergo processing that includes roasting under high temperatures [7]. During such processes, a number of reactions such as Maillard, Strecker’s and caramelisation occur at temperatures over 200 °C during roasting, which result in the characteristic aroma and bitter taste of the coffee beverage [8,32]. In the current study, sucrose content reduced by 97% when the green beans were roasted, due to its participation in the above reactions (Table 2). During roasting, free amino acids in the coffee beans react with fructose and glucose produced from sucrose digestion beforehand during fermentation to form a number of odorants such as 2–furfurylthiol, 2, 3 butanedione amongst others, whose identity is rather determined by the type of amino acid involved in the reaction [8,9]. Additionally, coffee flavour is also determined by pyrroles and pyridines from trigonelline degradation [9,10]. However, only a small fraction of trigonelline is involved in this reaction, hence minor reductions in the content between raw and roasted beans occurs, similar to what was observed in the current study (Table 2). On the other hand, coffee taste is determined by caffeine, which is thermally stable, and phenolic derivatives from chlorogenic acid degradation into melanoidins [11]. The content of 5–CQA dropped sharply when the beans were roasted, supporting their degradation into melanoidins and other related compounding at high temperatures (Table 2). However, the slight increase in caffeine in the roasted samples could have resulted from the differences in the moisture content when the GB were roasted.

Leaves are associated with high rates of metabolism due to their role in photosynthesis. For this, they contain chlorophylls to facilitate photosynthetic activity. The concentration of total chlorophylls in the leaves or proportions of their respective types (*a* and *b*) varies with leaf age or position. Chlorophyll *a* is normally highest in the youngest leaves, whereas chlorophyll *b* is highest in mature leaves. The latter is normally found in the reaction centres of photosystem I, II and in the pigment antenna system, whereas the former is found only in the pigment antenna system [27]. In the current study, there was a general increase in the concentration of the chlorophyll *b* in the older leaves (Table 3). It is suggested that this is meant to maximize light capture because of the quaternary arrangement of leaves on the orthotropic stem, which dictates older leaves receive less incident light than their younger counterparts [33]. Chlorophylls are normally unable to utilize all the photosynthetically active radiation (PAR) and, therefore, plants have evolved mechanisms to avoid or detoxify ROS that result from excess excitation energy. In addition to energy evasion, by accumulating less amounts of chlorophylls [34], leaves contain carotenoids that serve to protect the chlorophylls against oxidative stresses [19]. These pigments have also been reported to contribute to health benefits such as decreasing disease risk due to their high antioxidant activities when consumed [19,35]. The presence of high amounts of other phytochemicals such as alkaloids, phenolic compounds and sugars has also been reported in the coffee leaves [13,14,16]. Our results also showed similar findings, especially in the youngest leaves. Like carotenoids, these compounds protect the leaves against ROS that are by-products of aerobic metabolism, more so in the young leaves [36,37,38]. These compounds normally complement the enzymatic defence system in detoxifying the ROS [39]. It has recently been shown that unlike the older counterparts, young coffee leaves have a poorly developed enzymatic antioxidant defence system and hence the reliance on oxidant scavenger compounds is inevitable [40,41]. In addition to defence, some phytochemicals in the current study have other functions in coffee plants. Sucrose, a highly soluble disaccharide, is synthesised in the leaf cytosol, and hence its accumulation is directly related to photosynthesis [42]. By virtue of their position, the youngest leaves accumulated the highest content of sucrose, which reduced with leaf maturity (Table 3). It is also a storage reservoir molecule and a transportation solute, which is readily broken down to provide energy for growth and other cellular functions [43]. 

In the current study, relatively higher amounts of sucrose accumulated in the HS compared to roots, while WS had the least content (Table 2). This could be due to the presence of active meristems in both the HS and the roots that require the energy for growth [43]. Accumulation of phytochemicals such as caffeine, 5–CQA and mangiferin in other organs of the coffee plants like the stem (especially WS) and roots is less reported. Nevertheless, this study confirmed the presence of high amounts of sucrose and 5–CQA in the roots (Table 2). Although evidence of chlorogenic acid metabolism in the roots remains uninvestigated, their accumulation has been suggested to be because of their regulatory role in root hair formation [44]. Mangiferin and caffeine were essentially absent in the WS and the roots. This observation is in agreement with similar findings that have suggested that mangiferin, a bioactive xanthonoid compound, accumulates in the photosynthetic tissues so as to protect the organs against ultraviolet stress [14]. On the other hand, caffeine is known to protect against herbivory and therefore accumulates only in the forage tissues, especially leaves and beans and hence less in lignified tissues such as WS and roots [21]. Though in lesser amounts, trigonelline was present in the HS, roots and WS, in that order. This pyridine alkaloid accumulates in coffee organs as a reservoir for nicotinamide adenine dinucleotide (NAD) biosynthesis, which plays a key role in sub–cellular energy metabolism [4]. 

Biosynthesis of these phytochemicals is normally limited to specific organs. Phenolic compounds accumulation mainly occurs via the phenylpropanoid biosynthetic pathway [45]. However, just like in Campa et al. [14], this study found no correlation between chlorogenic acids (5–CQA) and mangiferin accumulation in the plant organs (Table S1). This is owed to the absence of metabolite competition for the two phenolic compounds and the silencing of the gene that encodes 3–ketoacyl–CoA thiolase (PhKAT1) protein, which catalyses the committed step for benzoic acid production in the benzenoid biosynthetic pathway [46] from which mangiferin biosynthesis proceeds. Moreover, unlike chlorogenic acids that are distributed in all organs of the coffee plant [22], recent reports have reported the presence of mangiferin only in the photosynthetic tissues of the coffee leaves and the receptacle of the young fruits of arabica coffee, which is in agreement with our findings [14]. The two phenolic compounds are however degraded during senescence, which could explain the decrease in the content of mangiferin and 5–CQA in BL (Table 2). On the other hand, alkaloids are metabolised in young leaves and the growing tips of the coffee plants and therefore accumulation of caffeine in older leaves is as a result of deposition rather than active biosynthesis where they are protective against herbivory [23]. Although it was earlier suggested that trigonelline also acts as a chemical defence against herbivory [47], recent reports have suggested that trigonelline biosynthesis results from detoxification of excess nicotinic acid and therefore is reconverted to the required substrate whenever the need for NAD biosynthesis arises [48]. Moreover, trigonelline accumulation was almost equally distributed in all the plant organs, especially those with active meristems. Our results agree with Ashihara and Watanabe [48] who have also reported presence of trigonelline in all coffee plant organs, with higher amounts especially in the upper stem and relatively lower amounts in the roots. Metabolism of the two alkaloid compounds occurs through two pathways, the *de novo* pathway and the salvage pathway. These two pathways for the alkaloids have been reported to occur simultaneously in the youngest buds and expanding leaves, hence resulting into high accumulation of alkaloids in such organs. On the other hand, the mature leaves contain only the salvage pathway, which is further constrained by reduced endogenous supply of the necessary substrates during biosynthesis [23]. Caffeine and trigonelline are degraded by demethylation into xanthine and nicotinic acid in mature plant organs. Our results suggest that caffeine degradation could be occurring at higher rates compared to that of trigonelline, hence a higher degradation percentage in BL due to loss in biological value in dried leaves [23,49]. The pattern of biosynthesis and accumulation of the two main alkaloid compounds, caffeine and trigonelline, in coffee seeds, especially the pericarp, follows a similar trend [4]. It has been reported that, largely, the two alkaloids are biosynthesized elsewhere and transported to the fruits and the seeds during maturation [4]. Therefore, the difference in caffeine and trigonelline content in the seeds corresponded with the difference in the youngest leaves, which are the main sites of alkaloid biosynthesis.

Coffee plants are an important source of dietary antioxidants. Antioxidant capacity of several foods including coffee is reported to be as a result of polyphenol accumulation [20], which include mangiferin and 5–CQA. The current study revealed that coffee leaves contained the highest of total phenolic content compared to other organs (Table 4). This could due to exposure of the leaves to oxidative stresses resulting from ultraviolet radiation and/or pathogens which the polyphenols protect against [50]. In coffee beans, roasting significantly increased the total phenolic content which could be due to thermal degradation of complex phenolic compounds such as chlorogenic acids into simpler ones like melanoidins with several hydroxyl components and glycosylic linkages [11]. As a consequence, the ROS scavenging capacity determined by DPPH, FRAP and ABTS was highest in the leaves, followed by beans, HS, roots, and least in WS. It is presumed that this order is dependent on the risk of ROS accumulation and hence an increase in total phenolic content. Moreover, Alvarez-Jubete et al. [20] also reported a strong positive correlation between TPC and oxidant scavenging capacity, while these parameters strongly negatively correlate DPPH IC_50_, as indicated in Figure 2. Phenolic compounds contain hydroxyl components and glycosylic linkages that scavenge ROS [40]. Antioxidant capacity and related benefits on human health are however dependent on bioavailability of the phytochemicals after consumption, which in turn is dependent on the soluble parts of the sample also known as extraction yield [5,16]. The results in the current study suggest that in addition to coffee beans, other coffee organs, especially the leaves, are also a major source of phytochemicals and bio–available antioxidant compounds. 

## 5. Conclusions

The phytochemicals determined in this study were generally distributed in all organs of the coffee plant in different amounts. Nevertheless, mangiferin was only present in the leaves, and caffeine was barely present in WS and roots. Although GB contained the highest content of the total phytochemicals analysed, coffee leaves contained higher amounts of the phytochemicals than RB. Leaves also contained mangiferin, chlorophylls and carotenoids which were absent in the both GB and RB. In addition, leaves contained the highest total phenolic content compared to other samples. Amongst other organs, relatively higher amounts of phytochemicals accumulated in HS than was present in roots, while WS had the least content. Of the phytochemicals that were studied, sucrose and 5–CQA were the main compounds followed by caffeine and trigonelline, while mangiferin was least in the organs when present. Due to thermal degradation of 5–CQA and reaction of sucrose during roasting, coupled with the relative stability of the alkaloids at high temperatures, it was revealed that caffeine and trigonelline were the main metabolites present in RB. Nevertheless, caffeine was more degraded during senescence compared to trigonelline and the other phytochemicals in the leaves. Accumulation of high amounts of phytochemicals in the leaves and beans resulted in the highest antioxidant capacities, as measured by DPPH, ABTS and FRAP assays in the respective samples. Other organs such as HS, roots and WS had the least antioxidant activities, in that order. Therefore, on the account of the phytochemicals, TPC and antioxidant capacity examined, coffee leaves might have the highest therapeutic and pharmacological value and, therefore, their utilization as a health beverage should not be ignored. Moreover, exploitation of coffee leaves will certainly add extra income to coffee farmers when materials arising from managerial practices such as de–suckering, in which new shoots are regularly pruned, can be economically utilised for “coffee leaf tea” processing. 

## Figures and Tables

**Table 1:** Time-course changes in the composition of the mobile phase during linear gradient analytical HPLC.

Time (min)	Methanol (100%)	Acetic Acid (2%)
0	15	85
4	15	85
10	35	65
22	43	57
24	60	40
28	60	40
30	15	85
40	15	85

**Table 2:** HPLC phytochemical profile of coffee plant organs expressed as unit weight per freeze-dried sample (mg g^−1^ DW).

Sample (Plant Organ)		Caffeine	Trigonelline	5–CQA	Mangiferin	Sucrose	5–CQA/Caffeine	HPLC Total
**Seeds**	GB	0.97 ± 0.09 ^b^^,c^	0.65 ± 0.05 ^a,b,c^	3.13 ± 0.33 ^a,b,c^	0.00 ± 0.00 ^c^	4.95 ± 0.40 ^a^	3.23 ± 0.03 ^c^	9.70 ± 0.73 ^a^
	RB	1.30 ± 0.13 ^a,b^	0.85 ± 0.01 ^a^	1.00 ± 0.02 ^e^	0.00 ± 0.00 ^c^	0.14 ± 0.05 ^g^	0.77 ± 0.07 ^c^	3.29 ± 0.20 ^g,h^
Leaves	L1	0.87 ± 0.03 ^c^	0.67 ± 0.22 ^a,b,c^	3.97 ± 0.61 ^a^	0.09 ± 0.03 ^a^	2.63 ± 0.55 ^b^	4.58 ± 0.85 ^c^	8.23 ± 0.18 ^b^
	L2	0.78 ± 0.13 ^c^	0.72 ± 0.26 ^a,b^	3.35 ± 0.37 ^a,b^	0.05 ± 0.01b	2.30 ± 0.16 ^b,c^	4.46 ± 1.26 ^c^	7.19 ± 0.15 ^b,c^
	L3	0.90 ± 0.37 ^c^	0.68 ± 0.20 ^a,b,c^	3.21 ± 0.24 ^a,b,c^	0.05 ± 0.01b	2.35 ± 0.34 ^b^	4.15 ± 2.10 ^c^	7.19 ± 0.43 ^b,c^
	L4	1.35 ± 0.07 ^a^	0.76 ± 0.12 ^a,b^	2.83 ± 0.23 ^b,c^	0.05 ± 0.00b	1.95 ± 0.08 ^b,c,d^	2.10 ± 0.06 ^c^	6.93 ± 0.20 ^c,d^
	L5	1.12 ± 0.00 ^a,b,c^	0.44 ± 0.03 ^b,c,d^	2.35 ± 0.11 ^c,d^	0.04 ± 0.01b	1.85 ± 0.16 ^b,c,d^	2.09 ± 0.09 ^c^	5.81 ± 0.06 ^d,e^
	L6	1.05 ± 0.01 ^a,b,c^	0.47 ± 0.01 ^b,c,d^	2.48 ± 0.30 ^b,c,d^	0.06 ± 0.02 ^a,b^	1.51 ± 0.12 ^c,d,e^	2.36 ± 0.28 ^c^	5.57 ± 0.46 ^e,f^
	BL	0.32 ± 0.00 ^d^	0.34 ± 0.04 ^c,d^	1.55 ± 0.32 ^d,e^	0.00 ± 0.00 ^c^	0.82 ± 0.12 ^e,f,g^	5.44 ± 0.67 ^c^	3.00 ± 0.42 ^h,i^
Stem	HS	0.01 ± 0.00 ^d^	0.45 ± 0.04 ^b,c,d^	2.67 ± 0.29 ^b,c^	0.00 ± 0.00 ^c^	1.24 ± 0.35 ^d,e,f^	190.2 ± 58.3 ^b^	4.37 ± 0.60 ^f,g^
	WS	0.00 ± 0.00 ^d^	0.20 ± 0.08 ^d^	0.66 ± 0.23 ^e^	0.00 ± 0.00 ^c^	0.57 ± 0.11 ^f,g^	369.4 ± 193.2 ^a^	1.42 ± 0.32 ^j^
Roots	Roots	0.00 ± 0.00 ^d^	0.14 ± 0.00 ^d^	0.71 ± 0.44 ^e^	0.00 ± 0.00 ^c^	1.01 ± 0.30 ^e,f^	0.00 ± 0.00 ^c^	1.85 ± 0.69 ^i,j^

Data are expressed as means ± S.D, *n* = 3. Within a column, (^a–j^) data means followed by the same letter are not statistically different by Tukey’s test (*p* ≤ 0.05). GB = green beans, RB = roasted beans, L1 to L6 = leaf pairs (1st—6th) from top to bottom of the coffee plant, BL = brown leaves of the first pair, HS = herbaceous stem, WS = woody stem.

**Table 3:** Concentration of chlorophylls and carotenoids in the leaves of coffee plants expressed as unit weight per fresh weight leaf (mg g^−1^ FW).

Leaf Position	Chlorophyll *a*	Chlorophyll *b*	Total Chlorophyll	Total Carotenoids	Chlorophyll *a*/*b*	Chlorophyll/Carotenoid
L1	1.09 ± 0.28 ^a^	0.37 ± 0.09 ^b^	1.46 ± 0.36 ^a^	0.26 ± 0.06 ^a^	2.93 ± 0.20 ^a,b^	5.48 ± 0.27 ^a^
L2	1.02 ± 0.06 ^a^	0.35 ± 0.05 ^b^	1.37 ± 0.09 ^a^	0.24 ± 0.02 ^a^	2.90 ± 0.26 ^a,b^	5.70 ± 0.02 ^a^
L3	1.31 ± 0.12 ^a^	0.48 ± 0.01 ^a,b^	1.79 ± 0.13 ^a^	0.31 ± 0.04 ^a^	2.73 ± 0.20 ^a,b^	5.84 ± 0.38 ^a^
L4	1.28 ± 0.17 ^a^	0.52 ± 0.03 ^a^	1.80 ± 0.19 ^a^	0.30 ± 0.04 ^a^	2.45 ± 0.24 ^b^	5.96 ± 0.22 ^a^
L5	1.27 ± 0.05 ^a^	0.40 ± 0.07 ^a,b^	1.67 ± 0.11 ^a^	0.37 ± 0.09 ^a^	3.20 ± 0.48 ^a^	4.72 ± 1.23 ^a^
L6	1.08 ± 0.02 ^a^	0.36 ± 0.01 ^b^	1.44 ± 0.02 ^a^	0.28 ± 0.01 ^a^	3.02 ± 0.09 ^a,b^	5.06 ± 0.14 ^a^

Data are expressed as means ± S.D, *n* = 3. Within a column, (^a,b^) data means followed by the same letter are not statistically different by Tukey’s test (*p* ≤ 0.05). L1 to L6 represents leaf pairs from top (youngest) to bottom (oldest) of the coffee plant.

**Table 4:** Total phenolic content (TPC) and antioxidant capacity of the coffee plant organs.

Sample (Plant Organ)		Yield (%)	TPC Content (mg g^−1^ GAE)	DPPH IC_50_ (µg. Sample mL^−1^)	DPPH	ABTS	FRAP
					TEAC (µmol Trolox g^−1^ Sample)		
Seeds	GB	25.8 ± 4.40 ^a,b^	29.3 ± 1.40 ^c^	146.8 ± 12.0 ^c^	199.7 ± 16.1 ^a,b,c^	220.7 ± 22.4 ^a,b^	974.2 ± 83.4 ^a,b^
	RB	36.4 ± 11.9 ^a^	35.4 ± 1.49 ^b,c^	87.0 ± 5.73 ^c^	337.0 ± 22.2 ^a^	325.5 ± 81.0 ^a^	1104.4 ± 323.3 ^a^
Leaves	L1	26.6 ± 0.23 ^a,b^	65.1 ± 14.1 ^a^	90.6 ± 12.1 ^c^	328.0 ± 43.9 ^a^	345.7 ± 53.3 ^a^	1097.4 ± 132.8 ^a^
	L2	24.2 ± 1.54 ^b^	66.7 ± 19.0 ^a^	94.0 ± 38.5 ^c^	302.5 ± 141.7 ^a^	323.8 ± 72.1 ^a^	1016.6 ± 271.2 ^a^
	L3	23.1 ± 0.53 ^b^	63.6 ± 10.2 ^a^	119.0 ± 34.7 ^c^	268.3 ± 78.3 ^a,b^	306.1 ± 92.3 ^a^	1024.2 ± 229.2 ^a^
	L4	22.5 ± 2.96 ^b^	66.4 ± 3.95 ^a^	115.3 ± 15.8 ^c^	258.2 ± 35.4 ^a,b^	267.0 ± 48.4 ^a^	969.1 ± 173.8 ^a,b^
	L5	18.1 ± 2.89 ^b,c^	72.0 ± 15.6 ^a^	125.5 ± 10.1 ^c^	228.7 ± 29.1 ^a,b^	238.9 ± 8.12 ^a,b^	618.6 ± 247.5 ^a,b,c^
	L6	18.9 ± 0.94 ^b^	71.5 ± 2.47 ^a^	132.6 ± 15.7 ^c^	223.4 ± 26.5 ^a,b^	208.8 ± 15.7 ^a,b^	624.1 ± 104.1 ^a,b,c^
	BL	25.6 ± 3.04 ^a,b^	60.4 ± 8.71 ^a,b^	99.8 ± 22.6 ^c^	304.6 ± 79.0 ^a^	294.5 ± 17.7 ^a^	755.1 ± 156.4 ^a,b^
Stem	HS	16.9 ± 0.55 ^b,c^	27.8 ± 2.57 ^c^	263.8 ± 10.5 ^c^	110.9 ± 4.42 ^b,c,d^	124.8 ± 1.42 ^b,c^	469.7 ± 13.0 ^b,c^
	WS	6.4 ± 0.27 ^c^	8.7 ± 1.17 ^c^	1403.1 ± 406.0 ^a^	21.9 ± 5.59 ^d^	24.9 ± 7.44 ^c^	110.0 ± 34.0 ^c^
Roots	Root	14.6 ± 3.16 ^b,c^	15.8 ± 3.43 ^c^	916.1 ± 370.4 ^b^	36.8 ± 18.6 ^c,d^	41.5 ± 21.2 ^c^	156.7 ± 85.6 ^c^

Data are expressed as means ± S.D, *n* = 3. Within a column, (^a–c^) data means followed by the same letter are not statistically different by Tukey’s test (*p* < 0.05). GB = green beans, RB = roasted beans, L1 to L6 = leaf pairs (1st–6th) from top to bottom of the coffee plant, BL = brown leaves of the first pair, HS = herbaceous stem, WS = woody stem.

## Supplementary Materials

The following are available online at https://www.mdpi.com/2076-3921/9/2/93/s1, Table S1: Pearson’s correlation coefficients amongst the measured parameters shown in Table 2, Table 3 and Table 4.
