# Supplementary material for: Phytochemical Profile and Antioxidant Capacity of Coffee Plant Organs Compared to Green and Roasted Coffee Beans
Source: Antioxidants (Basel). 2020 Jan 22;9(2):93. doi: 10.3390/antiox9020093 (PMC7070527; doi:10.3390/antiox9020093)
Supplement: Supplementary file 1 [file antioxidants-09-00093-s001.pdf]

Table S1: Pearson's correlation coefficients amongst the measured parameters shown in tables 2, 3 and 4.

	Yield	TPC	IC50	DPPH	ABTS	FRAP	Trigonelline	5 – CQA	Caffeine	Mangiferin	Sucrose	5 – CQA/Caffeine	HPLC Total	Chl a	Chl b	Total Chl	Total Car	Chl a/b
TPC	.35*																	
IC50	-.69**	-.69**																
DPPH	.76**	.75**	-.76**															
ABTS	.80**	.74**	-.79**	.94**														
FRAP	.84**	.57**	-.75**	.87**	.92**													
Trigonelline	.63**	.30	-.63**	.52**	.61**	.69**												
5 – CQA	.29	.63**	-.65**	.51**	.59**	.64**	.48**											
Caffeine	.58**	.58**	-.64**	.61**	.65**	.68**	.77**	.43**										
Mangiferin	.12	.76**	-.40*	.46**	.51**	.44**	.31	.71**	.48**									
Sucrose	.18	.17	-.36*	.17	.24	.41*	.34*	.69**	.32	.25								
5 – CQA/Caffeine	-.57**	-.54**	.57**	-.56**	-.57**	-.53**	-.39*	-.33	-.56**	-.34*	-.31							
HPLC Total	.39*	.50**	-.64**	.48**	.56**	.68**	.62**	.90**	.61**	.56**	.88**	-.44**						
Chl a	.07	.80**	-.43**	.42*	.48**	.40*	.36*	.64**	.61**	.88**	.24	-.38*	.55**					
Chl b	.08	.79**	-.43**	.42*	.48**	.41*	.39*	.63**	.61**	.86**	.24	-.37*	.56**	.99**				
Total Chl	.074	.80**	-.43**	.42*	.48**	.40*	.37*	.64**	.61**	.87**	.24	-.38*	.56**	.1.0**	.99**			
Total Car	.06	.77**	-.42*	.39*	.45**	.37*	.33*	.59**	.61**	.84**	.23	-.37*	.53**	.98**	.95**	.98**		
Chl a/b	.07	.79**	-.44**	.43**	.48**	.39*	.35*	.63**	.57**	.88**	.26	-.38*	.55**	.97**	.93**	.96**	.97**	
Chl/Car	.10	.81**	-.44**	.46**	.51**	.42*	.41*	.67**	.57**	.90**	.27	-.38*	.58**	.97**	.98**	.98**	.93**	.95**

*. Correlation is significant at the 0.05 level (2-tailed).

**. Correlation is significant at the 0.01 level (2-tailed).
